# The serendipitous origin of chordate secretin peptide family members

**DOI:** 10.1186/1471-2148-10-135

**Published:** 2010-05-06

**Authors:** João CR Cardoso, Florbela A Vieira, Ana S Gomes, Deborah M Power

**Affiliations:** 1 Centre of Marine Sciences (CCMAR), Universidade do Algarve, Campus de Gambelas, 8005-139 Faro, Portugal

## Abstract

**Background:**

The secretin family is a pleotropic group of brain-gut peptides with affinity for class 2 G-protein coupled receptors (secretin family GPCRs) proposed to have emerged early in the metazoan radiation via gene or genome duplications. In human, 10 members exist and sequence and functional homologues and ligand-receptor pairs have been characterised in representatives of most vertebrate classes. Secretin-like family GPCR homologues have also been isolated in non-vertebrate genomes however their corresponding ligands have not been convincingly identified and their evolution remains enigmatic.

**Results:**

*In silico *sequence comparisons failed to retrieve a non-vertebrate (porifera, cnidaria, protostome and early deuterostome) secretin family homologue. In contrast, secretin family members were identified in lamprey, several teleosts and tetrapods and comparative studies revealed that sequence and structure is in general maintained. Sequence comparisons and phylogenetic analysis revealed that PACAP, VIP and GCG are the most highly conserved members and two major peptide subfamilies exist; i) PACAP-like which includes PACAP, PRP, VIP, PH, GHRH, SCT and ii) GCG-like which includes GCG, GLP1, GLP2 and GIP. Conserved regions flanking secretin family members were established by comparative analysis of the *Takifugu*, *Xenopus*, chicken and human genomes and gene homologues were identified in nematode, *Drosophila *and *Ciona *genomes but no gene linkage occurred. However, in *Drosophila *and nematode genes which flank vertebrate secretin family members were identified in the same chromosome.

**Conclusions:**

Receptors of the secretin-like family GPCRs are present in protostomes but no sequence homologues of the vertebrate cognate ligands have been identified. It has not been possible to determine when the ligands evolved but it seems likely that it was after the protostome-deuterostome divergence from an exon that was part of an existing gene or gene fragment by rounds of gene/genome duplication. The duplicate exon under different evolutionary pressures originated the chordate PACAP-like and GCG-like subfamily groups. This event occurred after the emergence of the metazoan secretin GPCRs and led to the establishment of novel peptide-receptor interactions that contributed to the generation of novel physiological functions in the chordate lineage.

## Background

The evolution of the secretin family of brain-gut peptides remains enigmatic despite being some of the first endocrine factors ever identified. For example, in 1902 the ground breaking experiments of Bayliss and Starling with dog intestinal extracts set off the search for the active principal and, by 1961, secretin (SCT) had been isolated and sequenced [1]. Currently, 10 peptides belonging to the secretin family have been isolated in humans and include; SCT, vasoactive intestinal peptide (VIP), pituitary adenylate cyclase-activating polypeptide (PACAP), peptide histidine methionine (PHM), PACAP-related peptide (PRP), growth hormone-releasing hormone (GHRH), glucagon (GCG), glucagon-like peptide (GLP 1 and 2) and glucose-dependent insulinotropic peptide (GIP) [2-4]. Members of the secretin family share significant structural and conformational homology and their key metabolic and developmental functions in human make them of considerable pharmacological interest. Members of class 2 G-protein coupled receptors (a.k.a family B GPCRs), bind and are activated by the secretin family members (family B1 members or secretin family GPCRs) and specific peptide-receptor pairs have been identified in representatives of different vertebrate classes. Class 2 GPCRs is a larger family of receptors and also includes members of the metazoan adhesion (B2) and insect methuselah (B3) families and secretin family GPCRs (B1) are proposed to descend from the adhesion receptors prior to protostome-deuterostome divergence [5,6].

**Figure 1:**
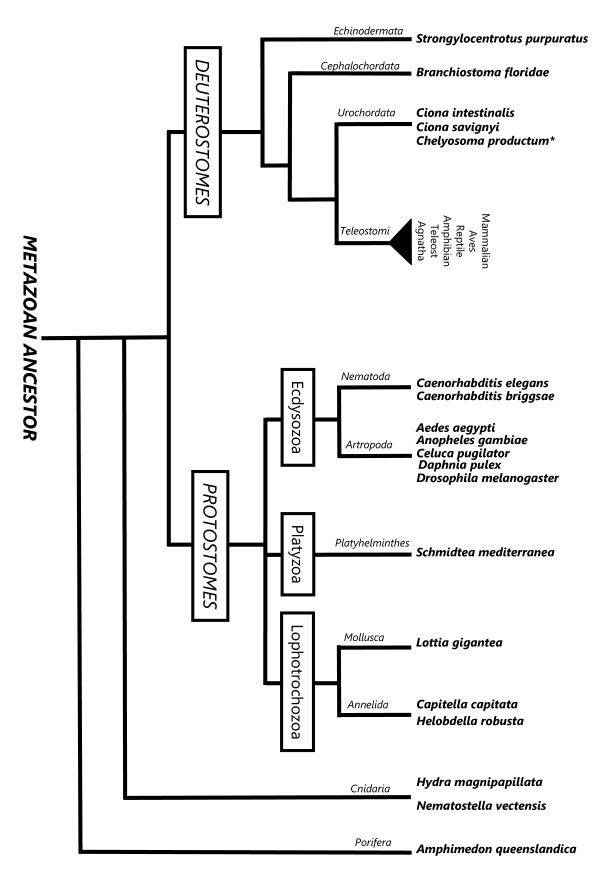
**Phylogenetic position of the non-vertebrate genomes analysed**. Simplified phylogeny of the metazoan evolution indicating the relative position of the early metazoa (Porifera and Cnidaria), protostome (Nematoda, Arthropoda, Platyhelminthes, Mollusca, Annelida) and early deuterostome (Echinodermata, Cephalochordata and Urochordata) genomes analysed (adapted from [96-98]). The tunicate *Chelyosoma productum *is also represented (*) since it is the only invertebrate in which secretin family members have been isolated [7].

In protostomes (nematodes, arthropods, annelids and platyhelminthes) and early deuterostomes such as *Ciona *and amphioxus, immunohistochemical (IHC) approaches using antisera raised against various mammalian secretin family members suggest they possess similar peptides to vertebrates (Table 1). PACAP-like genes (pacap1 and pacap2) have only been reported in the tunicate, *Chelyosoma productum *[7] and partial mRNAs (114bp) corresponding to the highly conserved PACAP coding exon [8] have been isolated in *Hydra magnipapillata *and several protostomes and deposited in public databases [9]. The existing data has been taken to indicate that an ancestral secretin family gene was probably present prior to the deuterostome-protostome divergence and most likely resembled the vertebrate PACAP precursor [2,8,10-12]. Paradoxically, in protostomes with fully sequenced genomes and extensive molecular resources (Figure 1, Additional file 1), genes encoding ligands homologous to members of the vertebrate secretin family have not been reported. In contrast, secretin-like family GPCR encoding genes which share similar sequence, structure and conserved gene environment with the vertebrate members have been identified, making ligand-receptor evolution an interesting enigma [13-16].

**Table 1:** Molecular and expression data available for the secretin members in metazoa.

	Species		*PRP/PACAP*	*PH/VIP*	*GHRH*	*GCG/GLP*	*GIP*	*SCT*	References
**DEUTEROSTOMES**									
VERTEBRATES									
Mammal	*Homo sapiens*		**N**	**N, P**	**N, P**	**N, P**	**N, P**	**N, P**	[100-109]
Ave	*Anas platyrhynchos*		**N**	**N**	**N**	**P**			[9,21,22]
	*Gallus gallus*		**N,P**	**N,P**	**N**	**N,P**	**N**	**P**	[24-27,35,110-112],
	*Meleagris gallopavo*			**N**		**N**			[23,113]
Reptile	*Alligator mississippiensis*			**P**		**P**			[28,29]
	*Heloderma suspectum*					**N**			[30]
	*Podarcis sicula*		**N**						[31]
Amphibia	*Rana catesbeiana*					**P**			[114]
	*Rana ridibunda*		**N,P**	**P**					[33,34]
	*Xenopus laevis*		**N**	**N**	**N**	**N**	**N**		[9,27,36-38]
	*Xenopus tropicalis*					**N**	**N**		[9,27],
Teleost	*Carassius auratus*			**N,P**	**N**	**N**			[37,41,45,115] ,
	*Clarias macrocephalus*		**N**			**N**			[9,116],
	*Danio rerio*		**N**	**N**	**N**	**N**	**N**		[9,27,37,47,83,117,118]
	*Gadus morhua*		**N**	**P**					[39,40]
	*Ictalurus punctatus*		**N**			**N,P**			[9,43,44]
	*Oncorhynchus nerka*		**N**						[119]
	*Takifugu rubripes*		**N**	**N**					[8]
	*Uranoscopus japonicus*		**P**						[42]
*Chondrichthyes*	*Torpedo marmota*		**N, IHC**						[120]
	*Dasyatis akajei*		**IHC**						[121]
	*Callorhynchus milii*					**P**			[122]
*Agnatha*	*Lampetra fluviatilis*					**P**			[123]
	*Lampetra japonica*		**N**						[9]
	*Petromyzon marinus*					**N,P**			[52,124]
UROCHORDATES	*Chelyosoma productum*		**N, IS**						[7]
	*Ciona intestinalis*			**IHC**				**IHC**	[125-127]
	*Halocynthia roretzi*		N*						[9]
	*Styela plicata*			**IHC**		**IHC**		**IHC**	[125,127-129]
CEPHALOCHORDATES	*Branchiostomata lanceolatum*			**IHC**		**IHC**		**IHC**	[127,130,131]
**PROTOSTOMES**									
NEMATODES	*Ascaridia galli*			**IHC**					[132]
	*Nematodirus battus*			**IHC**					[132]
	*Nippostrongylus brasiliensis*			**IHC**					[132]
ARTHROPODS	*Aeschna cyanea*			**IHC**					[133,134]
	*Eriocheir japonica*		N*						[9]
	*Eristalis aeneus*					**IHC**		**IHC**	[135]
	*Manduca sexta*			**IHC**		**IHC**	**IHC**	**IHC**	[136]
	*Periplaneta americana*		N*	**IHC**					[9,10,131,137,138]
ANNELIDS	*Hirudo medicinalis*			**IHC**					[131,139]
	*Lumbricus terrestris*		**IHC**	**IHC**					[131,140,141]
	*Nereis diversicolor*			**IHC**					[131,142]
	*Helix pomatia*			**IHC**					[143]
MOLLUSCS	*Mytilus galloprovencialis*			**IHC**					[144]
	*Planorbarius corneus*			**IHC**		**IHC**		**IHC**	[145]
	*Sepioteuthis lessoniana*		N*						[9]
	*Viviparus ater*			**IHC**		**IHC**		**IHC**	[146]
PLATYHELMINTHES	*Dugesia japonica*		N*						[9]
	*Schistosoma mansoni*			**IHC**					[147]
**CNIDARIA**	*Hydra magnipapillata*		N*						[9]

Nucleotide (N), Protein (P), Immunohistochemical (IHC) and *in situ *(IS)

N* non-vertebrate nucleotide sequences deposited in NCBI

The present study focuses on secretin family ligands and complements previous studies aimed at identifying and characterising the evolution of family 2 GPCRs [5,6,8,14,17]. A comparative approach which takes advantage of the wealth of information currently available (genome, ESTs, peptide) for porifera, cnidaria, protostomes, early deuterostomes and vertebrates (Figure 1), is undertaken to re-evaluate the origin of the secretin family in metazoa. The sequence, gene structure and gene environment of secretin family members in vertebrates with sequenced genomes was characterised and used to search for homologue peptides, genes or genome regions in non-vertebrates.

## Results

### Vertebrate secretin family members

Sequence database searches using the nucleotide and mature peptide sequences of human and zebrafish secretin family members, readily identified homologues in lamprey, teleost and tetrapod (*Xenopus*, lizard, chicken) genomes. This is due to the high sequence conservation of the mature peptide region between the vertebrate members which facilitates their identification (Table 2) (see reviews [2,4,8,18,19]).

**Table 2:** Accession numbers (ENSEMBL) of non-mammalian secretin members.

	PRP/PACAP	PH/VIP	GHRH	GCG/GLP	GIP	SCT
**Chicken ** *(Gallus gallus)*	ENSGALG00000014858	ENSGALG00000013604	ENSGALG00000003842	ENSGALG00000011104	ENSGALG00000001299	ENSGALG00000005081
**Lizard ** *(Anolis carolinensis)*	ENSACAG00000008729	ENSACAG00000005619	ENSACAG00000011836	ENSACAG00000014182	ENSACAG00000006291	----
**Xenopus ** *(Xenopus tropicalis)*	ENSXETG00000019179	ENSXETG00000027906	ENSXETESTG00000008409	ENSXETG00000013178	Scaffold_334	----
**Takifugu ** *(Takifugu rubripes)*	ENSTRUG00000003782 ENSTRUG00000010059	ENSTRUG00000001139	----	ENSTRUG00000008721 ENSTRUG00000004633	----	----
**Tetraodon ** *(Tetraodon nigroviridis)*	ENSTNIG00000017117 ENSTNIG00000018649	ENSTNIG00000007449	ENSTNIG00000007343	ENSTNIG00000013278 ENSTNIG00000000614	----	----
**Zebrafish ** *(Danio rerio)*	ENSDARG00000004015 ENSDARG00000027740	ENSDARG00000079443 ENSDARG00000078247	ENSDARG00000069481	ENSDARG00000042999/ ENSDARG00000079296 ENSDARG00000040907	ENSDARG00000071306	----
**Stickleback ** *(Gasterosteus aculeatus)*	ENSGACG00000017084 ENSGACG00000004163	ENSGACG00000001298	ENSGACG00000001298	ENSGACG00000013877 ENSGACG00000005606	----	----
**Medaka ** *(Oryzias latipes)*	ENSORLG00000017872 ENSORLG00000011205	ENSORLG00000003905	----	ENSORLG00000002782 ENSORLG00000016891	----	----
**Lamprey ** *(Petromyzon marinus)*	GENSCAN00000120210	GENSCAN00000109335/ GENSCAN00000056150	***----***	GENSCAN00000079364 Contig 32128	***----***	***----***

Contig/Scaffold numbers are indicated when ENSEMBL accession number are not available

Dashes indicate genes not identified

### The tetrapod members

In humans, 10 peptides encoded by six genes have been isolated. In *Aves*, homologues of the mammalian members have been identified and peptides and corresponding transcripts were isolated in duck (*Anas platyrhynchos*), chicken (*Gallus gallus*) and turkey (*Meleagris gallopavo*) [20-27]. *In silico *analysis of the chicken genome identified six genes encoding secretin family members which share similar organisation to the human homologues and comparative analysis revealed they correspond to the peptides and nucleotide precursors previously described (Table 1 and Table 2). Searches in the reptile and amphibia genomes identified homologues for all human members with the exception of SCT and it remains to be established if the failure is due to the incomplete nature of their genome assemblies or to the absence of this gene. In reptiles, few members of this family have been reported to date. A VIP and GCG peptides were isolated from the *Alligator mississippiensis*, the nucleotide precursor of the latter peptide reported from the *Heloderma suspectum *[28-30] and a PRP/PACAP mRNA was recently characterised from the Italian wall lizard *Podarcis sicula *[31,32]. Sequence database searches on the lizard *Anolis carolinensis *genome identified for the first time the genes encoding the reptile PHI/VIP (ENSACAG00000005619), PRP/PACAP (ENSACAG00000008729), GHRH (ENSACAG00000011836), GCG/GLP (ENSACAG00000014182) and GIP (ENSACAG00000006291) (Table 2). The predicted gene organisation suggests the coding exons for the mature peptides share identical structure with other metazoan genes and to date the exon encoding the lizard GLP2 remains to be identified.

Homologues of human secretin members have previously been reported in amphibians. VIP and PACAP and were isolated in *Rana ridibunda *[33,34]; GCG from *Rana catesbeiana *[35] and in *Xenopus laevis *single transcripts for PRP/PACAP [36], PHI/VIP, GHRH [37], GCG/GLP [38] and GIP [27] have been described. The genome of *Xenopus tropicalis *contains secretin family homologues which share high sequence conservation with the tetrapod genes (Table 2), but one difference was the presence of three GLP1 exons (GLP1a, b and c) within the GCG/GLP gene structure as a result of a species-specific exon duplication.

### The fish members

Peptides, transcripts and genes of the secretin family have also been isolated from the most diverse vertebrate clade, the teleosts. VIP was isolated from cod (*Gadus morhua*) [39,40] and goldfish (*Carassius auratus*) [41], PACAP from the Japanese stargazer (*Uranoscopus japonicus*) [42] and GCG from the channel catfish (*Ictalurus punctatus*) [43,44] (Table 1). The identification of two transcripts for PHI/VIP in goldfish *Carassius auratus *[45] and zebrafish (*Danio rerio*) (PHI/VIP *a*, EU031789 and PHI/VIP *b*, EU031790) and of two PRP/PACAP (PRP/PACAP *a*, NM_152885 and PRP/PACAP *b*, AF329633) [46,47] and GCG/GLP precursors in zebrafish [48] suggests they are duplicates in fish and this has been confirmed by the identification of two PRP/PACAP and GCG/GLP genes in *Takifugu *(ENSTRUG00000003782 and ENSTRUG00000010059; ENSTRUG00000008721 and ENSTRUG00000004633, respectively) and *Tetraodon *(ENSTNIG00000017117 and ENSTNIG00000018649; ENSTNIG00000013278 and ENSTNIG00000000614, respectively) genomes (Table 2) [8]. The greater number of secretin family genes identified in fish relative to tetrapods is most likely to be a result of the proposed teleost specific genome duplication and the absence of GIP and GHRH gene duplicates suggests they were probably deleted [49-51]. In common with *Xenopus*, no homologue of human SCT has been identified in fish genomes.

In the sea lamprey (*Petromyzon marinus*), a primitive vertebrate of the *Agnatha *clade, two homologues of human proglucagon (GCG/GLP) were characterised in the genome assembly Contig 31522 (GENSCAN00000079364) and Contig 32128 which correspond to the previously reported proglucagon I (AF159707) and proglucagon II (AF159708) transcripts, respectively [52]. In the present study, searches of the partially sequenced lamprey genome retrieved putative PRP/PACAP (GENSCAN00000120210 on Contig3575.2) and PHI/VIP genes (shared between GENSCAN00000109335 and GENSCAN00000056150 localised on Contig20045.2 and Contig20045.3, respectively). However, GHRH, SCT and GIP were not identified possibly due to the present incomplete nature of its genome assembly.

### Sequence and gene structure comparison

**Figure 2:**
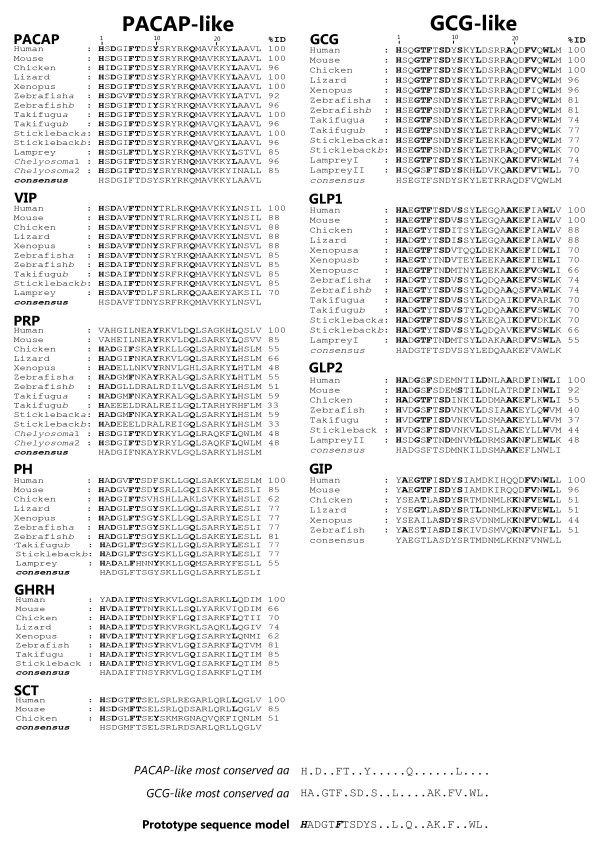
**Amino acid sequence conservation of vertebrate secretin family mature peptides**. The mature peptide sequences were extracted by comparison with the human homologues and only the amino acid (aa) residues 1 to 27 are represented with the exception of the first 5 residues of human GLP1 (P01275). *Takifugu *GHRH was obtained from [93] (N000079, Assembly_4) and the lamprey GLP2 sequence for proglucagon II was not used since it was found to share very little similarity with other vertebrate members suggesting it has undergone species-specific evolution. Vertebrate peptides are grouped according to their potential origin from a PACAP-like exon or GCG-like exon. Percentage of identity (%ID) for the human homologue is given and the consensus sequences for each peptide group were deduced using the GeneDoc programme [90] and used to generate a PACAP-like and GCG-like subfamily peptide. The most frequent residues within the different peptide groups are annotated in bold and a prototype model sequence for the chordate secretin family members was derived by fusing the conserved PACAP-like and GCG-like subfamily amino acid sequence (overlapping residues are annotated in bold and italics). Accession numbers of the teleost and non-mammalian sequences used are indicated in Table 2. The human precursors are PHM/VIP, P01282; PRP/PACAP, P18509; GHRH, P01286; GCG/GLPs, P01275; GIP, P09681; and SCT, P09683 and mouse (*Mus musculus*) accession numbers are PHM/VIP, P32648; PRP/PACAP, O70176; GHRH, P16043; GCG/GLPs, P55095; GIP, P48756; and SCT, Q08535.

Sequence comparisons reveal that members of the secretin family are highly conserved and this also applies to their secondary structure which consists of a random N-terminal structure and a C-terminal alpha-helix [53,54]. The lamprey, teleost, *Xenopus*, reptile and chicken members are in general 50% identical in amino acid sequence with the human homologues (Figure 2) and two main peptide subfamilies, which share in general a maximum of 60% sequence similarity between their members were identified; i) PACAP-like subfamily which includes 6 peptide groups (PACAP, PRP, VIP, PHI, GHRH and SCT) and ii) GCG-like subfamily which contains 4 peptide groups (GCG, GLP1, GLP2 and GIP). Highest sequence conservation from lamprey to human (>70% sequence identity) occurs within PACAP, VIP and GCG peptide groups which contrasts with PRP and GLP2 that are the most divergent and *Takifugu *PRP *b *and GLP2 predicted peptides are only 33% and 37% identical with the human homologues.

Consensus amino acids for peptide subfamilies were deduced and the overall conservation characterised. With few exceptions, 7 amino acid residues H^1^, D^3^, F^6^, T^7^, Y^10^, Q^16 ^and L^23 ^are the most abundant across the PACAP-like subfamily (Figure 2). The residues H^1^, D^3 ^and L^23 ^are present in all peptide groups with occasional exceptions in some taxa. However, the motif F^6^T^7 ^is absent from the vertebrate PRP sequences and is only present in tunicate PRP and the residue Q^16 ^is absent from the SCT mature peptide and Y^10 ^is only present in the chicken SCT homologue. This suggests that specific modifications occurred within the conserved core domain of the PACAP-like subfamily members and their functional significance remains to be explored. A similar comparison of the vertebrate GCG-like subfamily indicates that 15 amino acid residues H^1^, A^2^, G^4^, T^5^, F^6^, S^8^, D^9^, S^11^, L^14^, A^19^, K^20^, F^22^, V^23^, W^25^, and L^26 ^are generally maintained across the 4 peptide groups. Even if taxa variability is taken into consideration, the residues G^4^, F^6 ^and F^22 ^and the motifs S^8 ^D^9 ^and W^25^L^26 ^are in general maintained (Figure 2). Peptide specific variations include for GIP, H^1 ^which is replaced by Y, L^14 ^which is replaced by M or V (with exception of lizard) and A^19 ^which is replaced by K or Q. In the GCG mature peptide sequence A^2 ^is replaced by S and in GLP2 with the exception of chicken and *Takifugu *T^5 ^is replaced by S. Comparison of the metazoan PACAP-like and GCG-like subfamily consensus sequence revealed they overlap for the residues H^1 ^and F^6 ^which are key amino acids in secretin GPCR binding affinity [19,53,55,56]. This suggests that, after exon/chromosome duplication of their common ancestor exon, distinct evolutionary pressures within each subfamily occurred.

**Figure 3:**
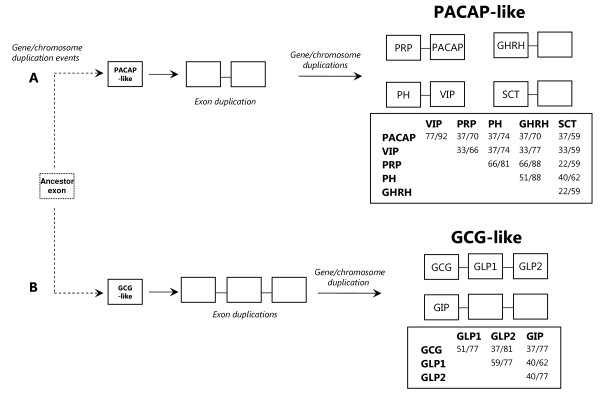
**Proposed evolutionary model of chordate PACAP-like (A) and GCG-like (B) members**. Percentage of amino acid sequence identity/similarity of the different peptide groups is indicated and gene organisation of the coding region (excluding occasional species-specific gene organisation) is represented. Secretin family members are proposed to have evolved via exon and gene/chromosome duplication events from a common ancestor exon in the chordate radiation. Similarity between the deduced consensus sequences of the peptide groups in the same subfamily is higher than 62% within the vertebrate GCG-like members and 66% for the PACAP-like subfamily with the exception of SCT in which only the mammalian and chicken members have been identified. Boxes represent exons and lines introns and coding exons are indicated by the peptide abbreviation. Dashed lines indicate undefined evolutionary pathways. (A) Chordate PACAP and PRP and vertebrate VIP and PH share the same gene precursor and GHRH and SCT are encoded by a single exon. PACAP and VIP share the highest amino acid conservation and SCT is the most divergent and to date has only been identified in tetrapods. (B) Vertebrate GCG, GLP1 and GLP2 are encoded in the same gene precursor which arose by exon duplication events. GIP is encoded by a single exon in a different precursor which has a similar gene organisation with GCG/GLP precursor.

The chordate PACAP shares in general 92% of amino acid sequence similarity with VIP and these two peptides are the most conserved members (Figure 3). In contrast, the vertebrate SCT demonstrates the lowest conservation (less than 62% within the PACAP-like subfamily) however this may be an artefact due to the restricted number of species in which it has been characterised. In addition, signature amino acid residues within or between peptide groups were also identified and they may reflect and support common evolutionary pathways and overlapping or specific functional roles. This includes, the PACAP Y^13 ^and A^24^A^25 ^motif, the V^5^, N^9^, F^13 ^and N^24 ^for VIP, the motif N^7^K^8^A^9 ^and residue H^24 ^within PRP, the L^5^, S^8^, E^24 ^and I^27 ^for PH, and the GHRH residues N^8^, I^17^, Q^24 ^and I^26 ^across the tetrapod and teleost members (Figure 2). Gene structure comparisons restricted to the mature peptide precursors revealed that PACAP and VIP exons are encoded in the same precursor as PRP and PH, respectively [2,8] (Figure 3). Most common amino acids to the chordate PACAP and VIP members includes R^14 ^and K^15^and the motifs M^17^A^18^V^19 ^and V^26^L^27 ^and they share a maximum of 37% amino acid sequence identity with PRP and PH indicating that after exon duplication considerable changes occurred. The latter peptides have a similar sequence (81% similar) which is closely related to vertebrate GHRH (88% similar) with which they partition the residues A^2^, K^12^, L^14^, G^15 ^and the motif S^18^A^19^R^20 ^and suggest a common evolutionary origin. The SCT gene has only been identified in tetrapods and the deduced peptide residues, E^9 ^and Q^20 ^are maintained in the mammalian and chicken homologues. The evolutionary origin of SCT is still enigmatic and it is proposed to have been lost in the fish lineage [14,57].

Members of the GCG-like subfamily share at least 62% amino acid similarity and GLP1 and GLP2 have the highest identity (59%, Figure 3). In addition to the general sequence conservation of the GCG-like subfamily members, conserved amino acid positions within the vertebrate peptide groups were also identified (Figure 2). This includes S^2^, Q^24^, M^27 ^and the motifs R^17^R^18 ^and Q^20^D^21 ^for GCG; V^10^, S^12 ^and Q^17 ^for GLP1; S^5^, N^11 ^and L^23 ^for GLP2; and Y^1 ^and L^27 ^in GIP. The GLP1 and GLP2 peptides are encoded in the same precursor (proglucagon precursor) as GCG with which they share 77% and 81% amino acid sequence similarity, respectively and the mature peptide coding exons are proposed to be a consequence of exon duplication events. Vertebrate GIP shares 37% and 40% sequence identity with GCG and GLPs, respectively and studies based upon gene structure comparisons suggest the latter emerged from the same exon as GCG, however the results of sequence analysis are inconclusive and more data is required [2,27].

### The secretin members in non-vertebrates

Database searches using the vertebrate nucleotide and peptide sequences of secretin family members and the duplicate urochordate *Chelyosoma productum *PRP/PACAP transcripts failed to identify conserved sequence and structure homologues in genome or EST databases from porifera, cnidaria, protostome and early deuterostomes (*Ciona*, amphioxus and sea urchin). Instead, short sequence matches were identified in unrelated genes or non-annotated genome segments. In depth analysis of the best matches, revealed homologies for the central region and C-terminal ends (outside the bioactive core) of the chordate mature peptides and, when the invertebrate fragments were used to interrogate vertebrate databases, they failed to retrieve a secretin family homologue suggesting that members of this family are absent from non-vertebrate genomes.

The exception is PACAP for which a highly conserved partial sequence corresponding to the exon encoding the mature peptide (>89% amino acid identity, [8]) has been isolated in *Hydra magnipapillata *(AB083650), in the tunicate, *Halocynthia roretzi *(AB121759) and in several protostomes such as planarian (*Dugesia japonica*, AB083649), crab (*Eriocheir japonica*, AB121765), squid (*Sepioteuthis lessoniana*, AB083651) and cockroach (*Periplaneta americana*, AB083652) [9]. Database searches using the non-vertebrate PACAP nucleotide or deduced peptide sequences failed to retrieve homologues from protostomes with available genome data (*Helobdella robusta, Capitella sp. I, Lottia gigantea, Daphnia pulex, Drosophila melanogaster*, *Aedes aegypti*, *Anopheles gambiae*, *Caenorhabditis elegans *and *Caenorhabditis briggsae*). Moreover, searches performed in *Hydra magnipapillata *and related species *Nematostella vectensis *genome assemblies with the *Hydra *PACAP nucleotide or deduced peptide sequence also failed to confirm the existence of a gene encoding PACAP. In early deuterostomes, searches using the *Chelyosoma productum *PRP/PACAP nucleotide or deduced peptide sequences in *Ciona intestinalis *and *Ciona savignyi *genomes or available tunicate ESTs failed to identify possible sequence homologues in urochordate. Taken together these results raise questions about the authenticity of the previously reported sequences.

An alternative strategy utilized a secretin family prototype sequence model based on the assumption that the chordate members arose from a common precursor gene which duplicated to give PACAP-like and GCG-like subfamily exons (Figure 2 and 3). The prototype sequence deduced *in silico *was H^1^A^2^D^3^G^4^T^5^F^6^T^7^S^8^D^9^Y^10^S^11^xxL^14^xQ^16^xxA^19^K^20^xF^22^xxW^25^L^26 ^(x represents variable position) and contains 18 conserved amino acid positions and high conservation was found for the N-terminal region. Structural characterisation using Pfam analysis classified the generated prototype sequence as a Hormone_2 member (PF00123, which includes the vertebrate secretin family members) [58] and sequence similarity searches performed retrieved secretin family members in vertebrates but failed to identify potential members in non-vertebrates. A second approach using HMM models and searching the general NCBI non-redundant (nr) peptide and an invertebrate subset of the NCBI nucleotide and EST (est_others) databases corroborated the preceding results.

### Phylogenetic analysis

**Figure 4:**
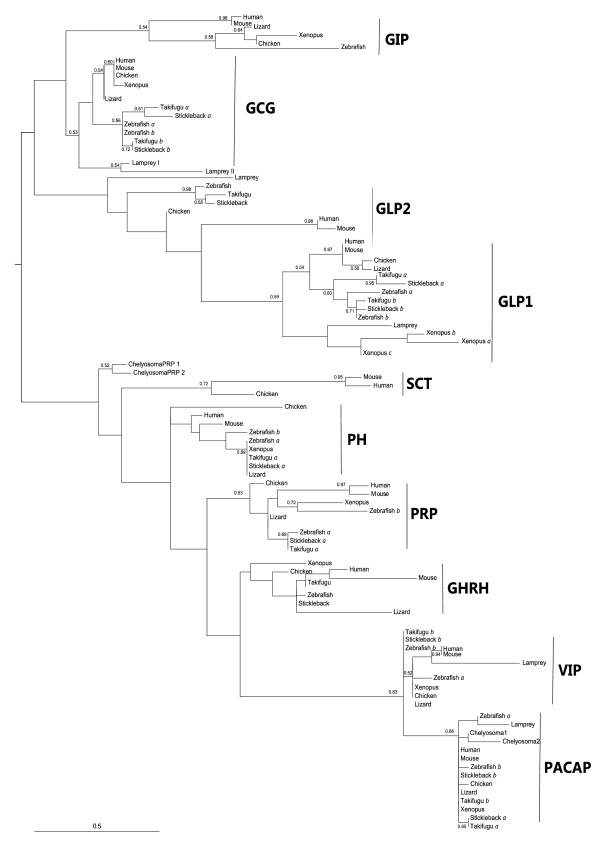
**Evolutionary analysis of the chordate secretin family members**. The maximum likelihood (ML) optimal tree topology is presented and was constructed with Phyml 3.0 [99]. ML bootstrap values higher than 50% are indicated at nodes and to facilitate interpretation a hypothetical root was added to the tree between the PACAP-like and GCG-like clades based upon gene structure evidence and proposed models for secretin family evolution. The different peptide groups are indicated and teleost duplicate genes are marked by *a *and *b*; *Xenopus *GLP1 exons by *a*, *b *and *c*. Accession numbers of the sequences used are described in Table 2 and for human and mouse members are: PHM/VIP (P01282 and P32648); PRP/PACAP (P18509 and O70176); GHRH (P01286 and P16043); GCG/GLPs (P01275 and P55095); GIP (P09681 and P48756); and SCT (P09683 and Q08535), respectively.

The optimal maximum likelihood (ML) tree with bootstrap support values higher than 50% is presented in Figure 4. Despite the high level of sequence identity and short sequences utilized, phylogenetic analyses of the chordate mature peptides (1-27 aa) and their corresponding nucleotide sequences resulted in similar tree topologies and suggests that members of the secretin family share a common ancestry. In the optimal ML tree presented in Figure 4 two clades PACAP-like and GCG-like were obtained suggesting that after ancestral exon duplication two main peptide subgroups emerged and underwent distinct evolutionary trajectories. Similar tree topologies were obtained using Bayesian approaches (Supplementary table 2) and a PACAP-like derived clade includes the peptides PACAP, PRP, PH, VIP, GHRH and SCT and a GCG-like clade the peptides GCG, GLP1, GLP2 and GIP. The *Chelyosoma *PACAP deduced peptide sequences always grouped with the vertebrate peptide sequence homologues and the tunicate PRPs did not cluster with any particular peptide clade and in all the analysis performed tended to be more closely related to the tetrapod SCT.

### Gene environment comparisons

**Figure 5:**
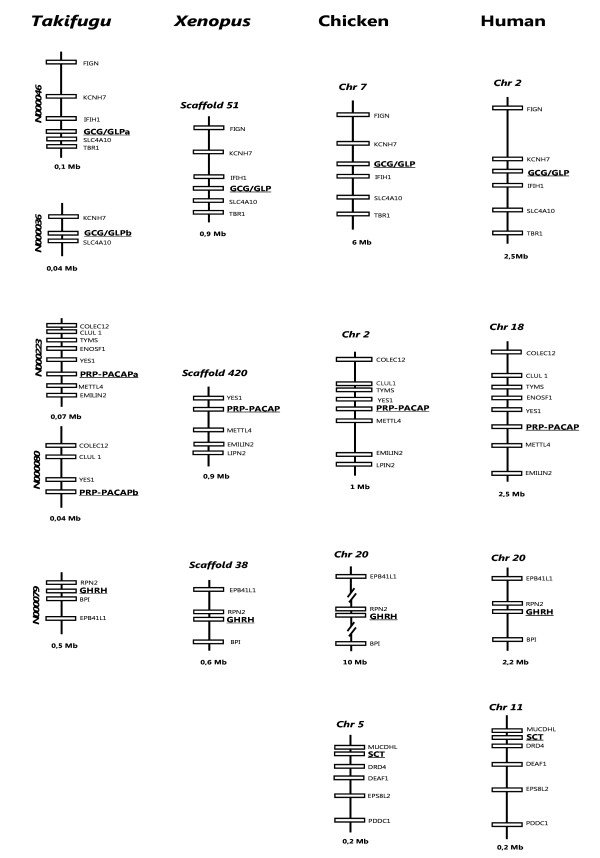
**Gene environment comparisons of the GCG/GLPs, PRP/PACAP, GHRH and SCT genes in *Takifugu*, *Xenopus*, chicken and human**. Homologue genes were identified using sequence similarity approaches with the *Takifugu *genes. *Takifugu *scaffolds are named according to the Assembly 4 available at [93] and have a direct correspondence with ENSEMBL (eg: N000046 corresponds to *Takifugu *Ensembl scaffolds_46). Genes were named based on HUGO annotation and the size of the genome regions analysed indicated within brackets. Genes are represented by boxes and genomic regions are indicated by lines. The figure is not drawn to scale and genes are positioned according to their relative distance in the genome assembly. For simplicity, only homologue genes are represented and GCG/GLP, PRP/PACAP, GHRH and SCT genes are edited in bold and underlined.

The immediate gene environment of vertebrate secretin members was compared and indicates that PRP/PACAP, GCG/GLP, and GHRH genome regions are syntenic and gene order is in general maintained (Figure 5). The GCG/GLP gene environment shares at least 3 genes (KCNH7, IFIH1 and SLC4A10), PRP/PACAP, 2 genes (YES1 and METTL4) and GHRH, 1 gene (RPN2) when equivalent genome regions are compared between *Takifugu*, *Xenopus*, chicken and human. The VIP and GIP genomic regions are poorly conserved and no gene synteny or gene order was identified (data not shown). Moreover, searches also failed to identify conservation of gene linkage between the different vertebrate secretin family members.

In *Takifugu*, the neighbouring genes of the paralogue GCG/GLP (SLC4A10 and KCNH7, N000046 and N000036) and PACAP (YES1, N000223 and N000080) genes were also duplicates supporting the teleost gene or genome duplication event. Searches were extended to the lamprey and lizard genomes but contiguous sequences were too small to confirm the existence of gene environment conservation. Comparison of the SCT genome region between chicken (chromosome 5) and human (chromosome 11) revealed they are highly conserved and the order of 5 genes (MUCDHL, DRD4, DEAF1, EPS8L2 and PDDC1) is maintained. In *Xenopus *genome, the chicken and human SCT flanking genes are localised in the amphibian scaffold_296 and in *Takifugu*, where a gene homologue is proposed to be absent, they are distributed in two distinct genome regions: MUCDHL and DRD4 are localised in scaffold N000002 and the remaining genes in scaffold N000328 suggesting the existence of a conserved gene block prior to the emergence of the tetrapod gene (data not shown).

**Figure 6:**
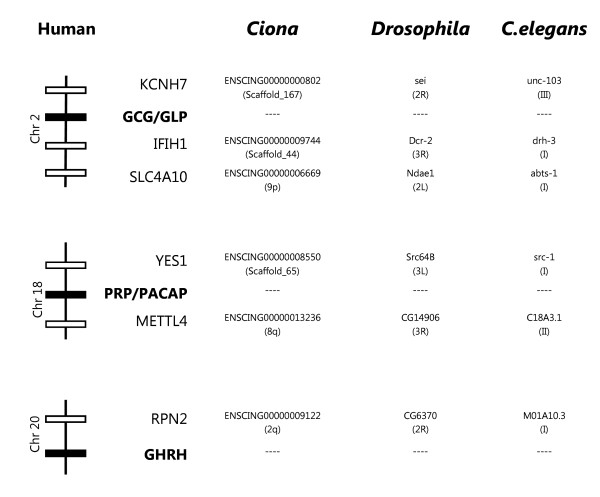
**Comparisons of conserved flanking genes of human PRP/PACAP, GHRH and GCG/GLP with the putative homologue regions in *Ciona*, *Drosophila *and *C. elegans***. Non-vertebrate genomes were accessed using the ENSEMBL annotation. Accession numbers of the human neighbouring genes: KCNH7 (EAX11346); interferon induced with helicase C domain 1 (IFIH1, EAX11352); Solute carrier family 4, sodium bicarbonate transporter, member 10 (SLC4A10, AAI36270), viral oncogene yes-1 homolog 1 (YES1, NP_005424), Methyltransferase like 4 (METTL4, AAI36768), ribophorin II, (RPN2, NP_002942).

In order to identify a potential secretin family genome region in non-vertebrates, the conserved vertebrate gene environment was used to retrieve homologues in the *C. elegans*, *Drosophila *and *Ciona *genomes. Genes sharing similarity in sequence to those flanking the vertebrate GCG/GLP, PRP/PACAP and GHRH loci were identified although the genes encoding secretin family members were absent (Figure 6). In *C. elegans *the genes *drh-3*, *abts-1, src-1 *and *M01A10.*3 map to chromosome I and are respectively homologues of the human *IFIH1 *and *SLC4A10 *on chromosome 2, *YES1 *on chromosome 18 and *RPN2 *on chromosome 20. In *Drosophila*, *sei *and *CG6370 *are localised in chromosome 2R and are the homologues of human *KCNH7 *and *RPN2 *and the fruit-fly *Dcr-2 *and *CG14906 *genes in chromosome 3R the correspondent in sequence of the human *IFIH1 *and *METTL*4, respectively.

In the tunicate genome, homologues of the vertebrate secretin family flanking genes were identified scattered in the *Ciona *genome assembly. Homologues of human KCNH7 (ENSCING00000000802, scaffold_167), IFIH1 (ENSCING00000009744, scaffold_44) and SLC4A10 (ENSCING00000006669, chromosome 9p) genes which flank GCG/GLP in vertebrates were identified. The conserved gene environment of the vertebrate PRP/PACAP genes: YES1 (ENSCING00000008550 on scaffold_65) and METTL4 (ENSCING00000013236 on chromosome 8q) genes are also present as well the putative urochordate homologue of the RPN2 gene (ENSCING00000009122) conserved in the vertebrate GHRH genome region.

In the *C. elegans *genome chromosome 1 contained homologues of the genes which flank PRP/PACAP, GCG/GLP and GHRH in vertebrates. This suggests that this chromosome may be the protostome genome fragment that most resembles the potential metazoan ancestral secretin genome region from which the vertebrate members emerged. However, detailed analysis of chromosome positions (*src-1*, 1566932 to 1580204bp; *M01A10.3*, 5550508 to 5549145 bp; *drh-3*, 7820837 to 7826373 bp; and *abst-1*, 8307558 to 8296909 bp) revealed they are not mapped in close proximity and do not forms a gene cluster. Moreover, no conserved linkage between the putative *Drosophila *and the *C. elegans *gene homologues exists.

## Discussion

Comparative analysis of data from phylogenetically distant organisms is a major contributor for understanding gene and gene family evolution and the role of function and regulation in this process. The identification of gene homologues in vertebrates and early metazoan genomes provides a unique opportunity to perform comparative studies and to investigate gene family ancestries. The secretin family is a well-studied group of peptides which activate specific receptors of family 2 GPCRs to bring about their pleotropic actions in vertebrates. Secretin-like family GPCRs have been identified and cloned from non-vertebrate genomes [6,14,59,60] and their putative peptide ligands identified by immunohistochemistry (IHC) using antisera raised against the mammalian peptide homologues (Table 1). Both peptides and their corresponding receptors are proposed to have arisen by gene duplication events prior to the vertebrate radiation [2,6,8,10,11,18] and they represent an interesting model for studies of receptor-ligand evolution.

In the present study, despite extensive *in silico *database searches it was not possible to identify members of the secretin peptide family in non-vertebrates regardless of the report of a full-length PACAP cDNA in the tunicate, *Chelyosoma productum *[7] and cDNA of the PACAP coding exon in crab, cockroach, squid, planarian and *Hydra magnipapillata *[7,9]. Moreover, using the partial PACAP cDNA previously identified in *Hydra magnipapillata *(a cnidaria) to interrogate its sequenced genome [61] failed to identify the gene homologue.

It seems unlikely that the failure to identify PACAP in non-vertebrates was related to the methodology used as a similar approach has been successfully utilized to identify the gene encoding the active nonapeptide hormone, vasotocin/vasopressin in the amphioxus genome assembly and also the gene loci in several chordates including teleosts [62]. In fact, the existence of neurohypophysial hormones (eg. vasopressin and oxytocin) in deuterostomes and protostomes has been amply confirmed by the isolation of both genes and peptides from representatives of a number of different phyla (reviewed in [62]). Similarly, conserved sequence homologues of vertebrate neuropeptide Y family members have been isolated from molluscs and also from fruit-fly and mosquito and GPCR ligand-receptor pairs similar to the vertebrate NPY system have been characterised [63-65].

A comprehensive *in silico *analysis of the fully sequenced *Drosophila *genome identified and classified GPCRs and compared their number to putative neuropeptide ligands. Although 5 secretin family GPCR members were identified in the arthropod genome, only two potential ligands, corticotrophin releasing factor (CRF)-related peptides and *amnesiac *genes, unrelated to chordate secretin peptide family members were predicted [66]. In contrast, recent studies performed in molluscs (*Helix pomatia*) using IHC and MALDI-TOF/TOF identified partial peptides with a similar mass to vertebrate PACAP in the snail whole hemolymph and CNS extracts [67]. Should the identity of the peptide be confirmed by sequencing taking in consideration the results of the present study a new paradigm will be required to explain secretin family evolution.

There is evidence that genes for *amnesiac *in *Drosophila *and maxadilan in sand-fly might encode functional homologues of the vertebrate PACAP despite their lack of sequence similarity [2,68-70] and the maxadilan peptide is able to activate mammalian PAC_1 _receptors *in vitro *[71,72]. The activation of family 2 GPCRs members by the secretin family of ligands has been linked to their well conserved structure [53,54] which comprises an alpha helix in the mid and C-terminal region and an N-terminal loop. The region of maxadilan implicated in PAC_1 _receptor activation has an identical structure to the N-terminal region of secretin family ligands and contains key amino acids involved in receptor activation (reviewed by [69]). In fact, mutation analysis with maxadilan demonstrated that despite its greater size compared to vertebrate secretin family peptides, the disruption of four conserved cysteine residues (1 - 5 and 14 - 51) responsible for the formation of two disulfide bonds led to loss of activity [69]. Taking into consideration the degree of conservation of the N-terminal ligand binding domain of the secretin family GPCRs [14] and their relative promiscuity [73] it is unsurprising that protostome peptides activate vertebrate receptors. In nematode and *Drosophila*, PDF (Pigment Dispersing Factor) stimulated the homologues of vertebrate secretin GPCRs but they were not stimulated by secretin family members [14,59,60]. These observations suggest that specificity of the receptor members has changed during evolution and may explain the failure to identify conserved ligands.

A general model to explain peptide ligand binding and receptor activation has emerged recently for class B GPCRs (secretin family) [74]. The proposed mechanism, known as the "two-domain model" suggests that initial ligand-receptor interactions are mediated by the central and C-terminal peptide segments with the extracellular N-terminal receptor region and that activation occurs subsequently when the bioactive N-terminus of the ligand binds the receptor juxtamembrane domain [75]. In this context, the identification in the present study of putative "signature" amino acids conserved across taxa for each peptide groups may explain differences in selection and affinity for receptors. For example, mutation of conserved residues within the C-terminal region of VIP revealed that substitution of L^23 ^(common to PACAP-like subfamily members) decreased peptide biological activity without altering the predicted structure [76]. D^3 ^is conserved across the PACAP-like peptides and this residue has a role in adenyl cyclase (AC) stimulation and interacts with basic residues (R^188^and K^195^) in the second transmembrane helix of VPAC [77]. Similarly, D^9 ^which is conserved across the GCG-like members is essential in the activation of mammalian GCGR [78]. It will be of interest in the future to study the role of unique amino acid residues/motifs identified in the present study within the N-terminal and C-terminal regions of each peptide group.

Recently, a Darwinian evolutionary model was proposed to explain the origin of steroid hormones and their receptors and may also explain the emergence of metazoan secretin peptide-receptor pairs. The steroid hormones and receptors were proposed to have evolved through a molecular exploitation process in which structurally adapted receptors evolved prior to ligand emergence [79,80]. By reconstructing the sequence of the ancestral steroid receptors (eg. corticoid receptor) the authors verified that they are activated by hormones (eg. aldosterone) that only emerged in the tetrapod lineage. At present, evolutionary comparisons of the metazoan receptor members and secretin peptide family suggests that, receptors emerged prior to the ligands which were subsequently acquired as a consequence of genome evolution in the chordate radiation. Generally, two major gene or genome duplication events are proposed to have occurred at the origin of vertebrates and have accompanied increased organismal complexity and emergence of gene novelties [81,82]. Whilst the majority of gene duplicates were probably lost as a consequence of their functional redundancy, some were fixed in the genome by the gain of new biological functions or partitioning the function of the ancestral counterpart and this may be the case for the secretin family GPCRs and their ligands [8,17,83].

The origin of the chordate secretin peptide family has been previously associated with the insect adipokinetic hormone (AKH) and AKH-Precursor Related Peptides (APRP) precursor evolution [84]. Despite their low sequence similarity, the arthropod AKH and APRP precursor was found to share a similar gene organisation and comparable functions with vertebrate GCG and GHRH and they were proposed to have shared common origin prior to protostome-deuterostome divergence (approximately 600 million years ago). However, against this hypothesis is the recent demonstration that insect ADK signals through a gonadotropin-releasing hormone (GnRH) like receptor (members of family 1 GPCRs) and also the isolation of putative nematode AKH-GnRH related precursors suggesting that the invertebrate ADK members may share common evolution with the metazoan GnRH system [85,86].

## Conclusions

The present study does not confirm the results of IHC studies in the early 70's and 80's which identified putative invertebrate secretin family members using antisera against mammalian peptides. It was not possible to identify sequence homologues of the *Chelyosoma productum *PRP/PACAP peptides in the sequenced *Ciona *genomes, although at least 8 putative secretin-like family GPCRs have been reported [14]. The previous facts taken with i) the identification in snail of a putative PACAP peptide; and ii) the activation of a secretin-like family GPCRs in nematode and *Drosophila *by PDF but not by vertebrate secretin family members makes it difficult to establish when the peptide members emerged in the deuterostome lineage. It is hypothesised that the emergence of the full suite of receptors and their ligands accompanied the rapid genome changes during chordate evolution. The ancestral secretin family gene probably arose as part of an existing gene or gene fragment and via exon and gene duplication events generated the existing suite of family members (Figure 3). This occurred after the emergence of the secretin family GPCRs and led to the establishment of novel and specific receptor ligand interactions that contributed to the generation of novel physiological functions. In contrast, to other peptide families, such as NPY/PYY and Oxytocin/vasopressin which stimulate receptors of family 1 GPCRs and are highly conserved from protostomes to deuterostomes, members of the secretin family GPCRs appear to have adopted new ligands during evolution.

## Methods

### Data mining

Using comparative sequence approaches the existence of putative non-vertebrate secretin family members were investigated in publicly available protostome and early deuterostome genome, EST and protein databases. The complete nucleotide and amino acid sequences of human secretin family members (PHM/VIP, P01282; PRP/PACAP, P18509; SCT, P09683; GHRH, P01286; GIP, P09681; and GCG/GLP, P01275) mature peptides and their homologues in zebrafish and tunicate *Chelyosoma productum *PRP/PACAP precursors were used to interrogate databases (Figure 1, Additional file 1). Searches were performed in the metazoan genomes of porifera (*Amphimedon queenslandica*), cnidarians (*Nematostella vectensis *and *Hydra magnipapilata*), planarian (*Schmidtea mediterranea*), annelids (*Helobdella robusta *and *Capitella capitata*), mollusc (*Lottia gigantea*), crustacean (*Daphnia pulex*), insects (*Drosophila melanogaster*, *Aedes aegypti *and *Anopheles gambiae*); nematodes (*Caenorhabditis elegans *and *Caenorhabditis briggsae*); and in the early deuterostomes, sea urchin (*Strongylocentrotus purpuratus Build 2*), cephalochordate (*Branchiostoma floridae*) and urochordates (*Ciona savignyi *and *Ciona intestinalis*); and also in the vertebrate sea lamprey (*Petromyzon marinus*), teleosts (zebrafish, *Danio rerio*; *Takifugu rubripes*; *Tetraodon nigroviridis*; medaka, *Oryzias latipes*; stickleback, *Gasterosteus aculeatus*) and tetrapods, frog (*Xenopus tropicalis*), lizard (*Anolis carolinensis*) and chicken (*Gallus gallus*). Searches of the *Takifugu *genome were also performed in http://fugu.nimr.mrc.ac.uk/blast. To substantiate the results, further searches for secretin family members were also carried out in the NCBI EST data sets for porifera (Porifera (taxid:6040), cnidaria (Cnidaria (taxid:6073), protostomes (Protostomia (taxid:33317)) and early deuterostome (Echinoderms (taxid:7586); Cephalochordata (taxid:7735); Urochordata (taxid:7712) and also in species specific EST databases for the planarian (*Schmidtea mediterranea*), crab (*Celuca pugilator*), pacific oyster (*Crassostrea gigas*) and mussel (*Mytilus edulis*) (Additional file 1). In addition, the complete nucleotide precursor or the sequence corresponding to the deduced mature peptide of secretin family members in deuterostome were used to interrogate the general nucleotide (nr/nt) and protein databases (nr) available at NCBI [9] and UniProt [87] using the BLAST programme.

For small mature peptide sequences the BLAST algorithm was adjusted (scoring matrix PAM30, word size 2, highest expected value parameters, low complexity filter off, no adjustment) to permit identification of short peptide hits with strong similarities. Searches using short nucleotide sequences were also performed with word size 7; expected value 1000 and low complexity filter off. Best matches with significant scores or low E values <0.01 were retrieved and analysed.

Searches using a hidden Markov model were performed with the HMMER3 (3.03b) [88] suite of software on the NCBI non-redundant (nr) peptide, and custom-made invertebrate nucleotide (nt) (1614126 records) and est_others (ests minus human and mouse) (11209486 records) databases using hmmsearch. Subset databases were constructed to reduce the computational burden of performing a HMMER3 search against the complete NCBI nucleotide and EST databases. The invertebrate subset databases were constructed by querying the NCBI databases using Entrez for all invertebrate GI numbers (Metazoa NOT Vertebrata) for both nt and est_others and filtered using fastacmd (part of the NCBI BLAST package). Peptide searches were performed with the Pfam model for the secretin peptide family members (Hormone_2 member, PF00123) and nucleotide queries with a model constructed in HMMER3 (3.03b) using the nucleotide aligned sequences of the 1-27 aa mature peptide regions of the secretin family members represented in Figure 2.

### Sequence comparisons

The potential secretin family members identified were compared with existing vertebrate members. The non-vertebrate sequences that shared similarity with previously annotated genes or gene intron regions were discarded and the remaining candidates used to interrogate the NCBI database to confirm identity and failed to retrieve a homologue of the vertebrate secretin family. In contrast, homologues in vertebrate datasets were identified and the deduced amino acid sequences of the retrieved transcripts or predicted exon coding regions were compared using ClustalX 2.0 [89] with the conserved mature peptide region 1-27 aa of representatives of the secretin family with the exception of human GLP1 in which 5-32 aa were used that correspond to a unique coding exon. Peptide similarities/identities were determined using the GeneDoc programme [90] and amino acid consensus sequences within each peptide group were deduced and compared to demonstrate general levels of conservation for each subfamily. A prototype peptide representative of the chordate secretin family was constructed by fusing the most abundant amino acid residues within the PACAP-like and GCG-like peptides members. The *in silico *deduced sequence was submitted to Pfam [58] analysis to confirm identity as a secretin member and used to search the vertebrate and non-vertebrate NCBI databases with BLAST and adjusted parameters to identify homologues.

### Phylogenetic analysis

Phylogenetic analyses were performed using the ClustalX 2.0 alignment of the 1-27 aa mature peptide region of secretin members. The amino acid sequence alignment produced was analysed with PROTTEST to select the model of protein evolution that best fits dataset [83] and phylogenetic analyses were conducted using 95 taxa with the maximum likelihood and Bayesian estimation methods (MrBayes and PhyloBayes, Additional file 2). The maximum likelihood analysis was carried out using Phyml 3.0 [85] with 100 bootstrap replicates with a JTT substitution model with a discrete gamma distribution of rates among sites with 4 categories (Г). A search for the optimal ML tree was also performed. Bayesian estimation using MrBayes [91] was performed with the Dayhoff model with Г and PhyloBayes [92] with the CAT model plus Г. The MrBayes analysis was conducted with two MCMC runs (each with 4 chains) for 200,000 generations with 20,000 samples. The PhyloBayes CAT analysis was performed using 2 independent run replicates (40727 and 40382 generations, respectively). Likelihoods were plotted against generation time and the MCMC chains were assumed to have reached stationarity when the curve plateaued. Phylogenetic sequence analysis was also performed using the nucleotide sequences of the 1-27 mature peptide domains with the ML method as previously described and the GTR model plus Г and the individual peptide clades present in both nucleotide and amino acid trees obtained were similar (data not shown).

### Gene environment comparisons

The gene environment of vertebrate secretin family members was determined to identify potentially conserved gene blocks; these were then used to search for putative ancestral secretin genome-like regions in *C. elegans*, *Drosophila *and *Ciona *assemblies. The *Takifugu *NIX annotated scaffolds [93] were used as a guide to characterise the *Xenopus *(*Xenopus tropicalis) *[94], the chicken and human homologue regions [95]. The NIX annotated scaffolds from *Takifugu *were used as they had greater information content than the homologue data deposited in ENSEMBL. The conserved vertebrate gene environment identified the genes YES1 and METTL4 for PRP/PACAP; the gene RPN2 for GHRH; and the KCNH7, IFIH1 and SLC4A10 genes within the vertebrate GCG/GLP genomic region. The, *C. elegans*, *Drosophila *and *Ciona *genomes were assessed using the ENSEMBL assembly annotation and homologues identified and compared with the vertebrate homologue region.

## Abbreviations

VIP: (Vasoactive Intestinal Peptide); PACAP: (Pituitary Adenylate Cyclase-Activating Polypeptide); SCT: (Secretin); GHRH: (Growth Hormone Releasing Hormone); PRP: (PACAP-Related Peptide); PH: (Peptide Histidine); PHI: (PH-Isoleucine); PHM: (PH-Methionine): GCG: (Glucagon); GLP: (GCG-like); GIP: (Glucose-dependent Insulinotropic Peptide); GPCRs: (G-Protein Coupled Receptors).

## Authors' contributions

The majority of the practical work was carried out by JCRC in collaboration with FAV and ASG. JCRC and DMP planned the study, wrote, analysed the data and critically revised the manuscript for important intellectual content. All authors read and approved the final manuscript.

## Supplementary Material

Additional file 1 Publicly available genome and EST databases searched using the nucleotide and mature peptide sequence of the tunicate (*Chelyosoma productum) *and human secretin family members. Click here for file

Additional file 2 **Phylogenetic support values (bootstrap proportions, bp; and posterior probabilities, pp) of the secretin family peptide clusters constructed with maximum likelihood (ml) (Figure**4)**, MrBayes and PhyloBayes methods.** Click here for file
