# Supplementary material for: The serendipitous origin of chordate secretin peptide family members
Source: BMC Evol Biol. 2010 May 6;10:135. doi: 10.1186/1471-2148-10-135 (PMC2880984; doi:10.1186/1471-2148-10-135)
Supplement: Additional file 1 — Publicly available genome and EST databases searched using the nucleotide and mature peptide sequence of the tunicate (Chelyosoma productum) and human secretin family members. [file 1471-2148-10-135-S1.PDF]

Additional file 1:

Publicly available genome and EST databases searched in this study.

	Specie	Genome/EST databases
**DEUTEROSTOMES**		
**Vertebrates**		
Aves	*Gallus gallus*	http://www.ensembl.org/
Reptile	*Anolis carolinensis*	http://pre.ensembl.org/
Amphibia	*Xenopus tropicalis*	http://www.ensembl.org/
Teleost	*Danio rerio*	http://www.ensembl.org/
	Gasterosteus aculeatus	http://www.ensembl.org/
	Oryzias latipes	http://www.ensembl.org/
	Takifugu rubripes	http://fugu.biology.qmul.ac.uk/; http://www.ensembl.org/
	Tetraodon nigroviridis	http://www.ensembl.org/
Agnatha	*Petromyzon marinus*	http://pre.ensembl.org/
**Urochordates**	*Ciona intestinalis*	http://www.ensembl.org/
	Ciona savignyi	http://www.ensembl.org/
**Cephalochordates**	*Branchiostoma floridae*	http://genome.jgi-psf.org/
**Echinoderme**	*Strongylocentrotus purpuratus*	http://www.hgsc.bcm.tmc.edu/projects/seaurchin/; http://goblet.molgen.mpg.de/
**PROTOSTOMES**		
**Nematodes**	*Caenorhabditis briggsae*	http://www.ensembl.org/
	Caenorhabditis elegans	http://www.ensembl.org/
**Arthropods**	*Aedes aegypti*	http://ensembl.org/
	Anopheles gambiae	http://ensembl.org/
	Celuca pugilator	http://www.genome.ou.edu/crab.html
	Daphnia pulex	http://genome.jgi-psf.org/
	Drosophila melanogaster	http://ensembl.org/
**Annelids**	*Capitella sp. I*	http://genome.jgi-psf.org/
	Helobdella robusta	http://genome.jgi-psf.org/
**Molluscs**	*Crassostrea gigas*	http://est.molgen.mpg.de/FishShellfish
	Lottia gigantea	http://genome.jgi-psf.org/
	Mytilus edulis	http://est.molgen.mpg.de/FishShellfish
**Platyhelminthes**	*Schmidtea mediterranea*	http://planaria.neuro.utah.edu/
**CNIDARIA**	*Hydra magnipapillata*	http://www.compagen.org
	Nematostella vectensis	http://www.compagen.org
**PORIFERA**	*Amphimedon queenslandica*	http://www.compagen.org
