# Supplementary material for: The serendipitous origin of chordate secretin peptide family members
Source: BMC Evol Biol. 2010 May 6;10:135. doi: 10.1186/1471-2148-10-135 (PMC2880984; doi:10.1186/1471-2148-10-135)
Supplement: Additional file 2 — Phylogenetic support values (bootstrap proportions, bp; and posterior probabilities, pp) of the secretin family peptide clusters constructed with maximum likelihood (ml) (Figure4), MrBayes and PhyloBayes methods. [file 1471-2148-10-135-S2.PDF]

Additional file 2:

Phylogenetic support values of the secretin family peptide clusters.

	Peptide Clusters	ml (bp)	MrBayes (pp)*	PhyloBayes (pp)#
GCG-like subfamily	GCG	0.54	<0.50	<0.50
	GIP	0.53	1.00	0.85
	GLP1	0.59	0.56	0.58
	GLP2	<0.50	0.71	0.66
PACAP-like subfamily	PACAP	0.66	0.80	<0.50
	VIP	0.83	<0.50	0.64
	GHRH	<0.50	0.99	0.93
	PH	<0.50	<0.50	0.58
	PRP	0.53	0.93	0.71
	SCT	0.72	0.98	0.58

boostrap proportions (bp); posterior probabilities,(pp)

* For the MrBayes analysis 10,000 trees were discarded as "burn-in" and a 50% majority-rule consensus tree was constructed with 10,000 trees assumed to have been drawn from the posterior probability distribution (-log Ln =-1876.58).

# In each of the two PhyloBayes MCMC analyses 10,000 samples were discarded as "burn-in" and sampled every 4th generation. The two chains were assumed to have converged to the same posterior distribution (maximum difference in clade PP 0.0764). A consensus tree was constructed by combining the samples from each chain (7,681 and 7,595 samples respectively).
